# The practicality of practice: A model of the function of play behaviour

**DOI:** 10.1002/ece3.10521

**Published:** 2023-09-19

**Authors:** Dave E. W. Mallpress, Marek Špinka

**Affiliations:** ^1^ Faculty of Agrobiology, Food and Resources Czech University of Life Sciences Prague Czechia

**Keywords:** dynamic programming, modelling, play behaviour, practice hypothesis

## Abstract

The function of play has been a long‐debated topic in animal behaviour. One popular class of accounts is that play offers practice for serious adult behaviour, but little has been done to model the circumstances where this could be true. In this paper, we model an individual who, over the juvenile and subadult ontogenetic periods, has a choice between three behaviours: foraging, playing and rest, where playing improves an individual's ability in some component of a serious adult behaviour. Using stochastic dynamic programming, we show that even when play is more energetically costly and an inferior form of practice than foraging itself, it still may be optimal to play under a variety of circumstances. We offer several instantiations of the play as practice concept to show the possibility of play improving a variety of different adult abilities: antipredatory, foraging and reproductive behaviour. These models show the environmental conditions where play might be expected, as well as the predicted occurrences of play throughout ontogeny. This is a first step in showing the ecological feasibility of the practice hypothesis of play and raises further questions about why playful activity is more beneficial than more deliberate directed practice.

## Graphical abstract

It has been hypothesised that play offers practice for serious adult behaviour. By modelling the repeated choices between three behaviours: foraging, play and rest, we show that play may be optimal, because of potential safety benefits, even if it only offers an inferior form of practice for serious behaviour. The model is applied to the practice of a variety of serious behaviours and subsequently predicts play for different periods in ontogeny.

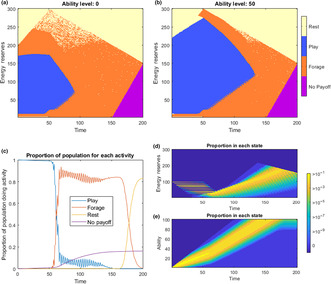

## 1 INTRODUCTION

Play is in stark contrast to other types of behaviours, which are normally defined by their function – foraging and eating maintain energy balance, courtship and mating lead to reproduction, and anti‐predatory behaviour leads to decreased risk of being killed for food. Moreover, in these cases of ‘serious’ behaviour, the causal link between the activity and its consequences is most often evident and recognisable. Play, on the other hand, is not defined by its consequences, but usually partly by the absence of them (Bekoff & Byers, 1981; Burghardt, 2005). One effect of this is that the approach to modelling the functional benefits of play will involve begging the question, by implicitly assuming those plausible benefits. Here, this research focusses on whether we can find circumstances in which play is not in any obvious way immediately beneficial and is clearly costly in some respects, but still predicted by an optimal model of lifetime behaviour.

The problems presented in forming accounts of play are amplified by the diversity of types of play and the richness and variety of actions, even within a single bout (McDonnell & Poulin, 2002; Petrů et al., 2009). Whether play is a useful unitary category of activity or a bundle of unrelated behaviours with similar characteristics remains an open question (Bateson, 2010; Pellis et al., 2019) and numerous plausible accounts of the function of play have been proposed over the past century (for more thorough reviews, see Baldwin & Baldwin, 1977; Bateson, 2010; Burghardt, 2005; Fagen, 1981; Gray, 2019). Some of the more prominent ultimate explanations for the existence of play include as a means of social bonding and establishing cooperation (Cordoni, 2009; Palagi, 2023), as a form of physical exercise (Brownlee, 1954), to assess one's abilities (Thompson, 1998), to train for unexpected situations (Špinka et al., 2001) and a source of innovation (Bateson & Martin, 2013). There are also common alternative accounts with a more limited functional basis, in particular, as a way to expend excess energy (the surplus resource model, Burghardt, 2005; surplus energy hypothesis, Barber, 1991). Given the diversity of species that exhibit play behaviours, and the variety of contexts and forms of play, it is not clear that all types of play can be accounted for by the same functional account. For example, if play improves social cohesion, acts of solitary play will remain unaccounted for (Petrů et al., 2009). Our approach in this article focuses on one popular class of explanations.

Perhaps the most common functional account of play is that it is a form of practice. This idea, arising from the fact play actions often closely resemble actions employed in various categories of ‘serious’ behaviour, was first formally proposed by Karl Groos, stating that play is “instinctive activity exerted for purposes of practice or exercise, and without serious intent” (Groos, 1895/1898, p.183). Over the last century, this idea has reappeared and been repeated in various forms, retaining the essence of ‘play as practice’, and only differing in the details of the hypothesis (Brownlee, 1954; Byers, 1998; Byers & Walker, 1995; Fagen, 1976a; Smith, 1982). Others have equated play with exploration, implying the latent learning functions of the activity (Baldwin & Baldwin, 1977; White, 1959). Various ‘alternative’ hypotheses of play retain the essential characteristics of practice, but capturing more general learned abilities, such as the ‘training for the unexpected’ hypothesis (Špinka et al., 2001). In this, individuals are not practising for a specific motor task, but rather for the ability to handle novel and unexpected situations, across the physical, cognitive, and emotional domains. Even some of the hypothesised functions relating purely to social play and its importance for social bonding, cooperation, or fairness, could be reinterpreted as a form of practice for important adult social skills that facilitate successful navigation of the adult social world (e.g. Allen & Bekoff, 2005; Pellis et al., 2010; Smith, 1982). Indeed, distinct ‘play behaviour systems’, each derived from a different category of serious behaviour and each training a different skill or ability, may exist in parallel even within the same species (Caro, 1995; Pellis et al., 2019).

Some good empirical support for hypotheses within the category of practice exists (e.g. Berghänel et al., 2015; Carter et al., 2019; Heintz et al., 2017; Nunes et al., 2004) although some studies have failed to find any effects at all (Caro, 1980; Sharpe, 2005). There is also plenty of empirical evidence for various other functional accounts of play behaviour (e.g. Nunes & Monroy Montemayor, 2023; Palagi et al., 2004; Pellis et al., 2010; Stark et al., 2021). However, there are difficulties in obtaining reliable long‐term, in‐the‐wild data of what are often probably delayed, relatively weak, and complex beneficial effects. Even when these long‐term beneficial effects are found (e.g. Fagen & Fagen, 2004; Nunes, 2014), it remains unclear what mechanisms directly link play to survival. The search for the functional benefits of play may be hampered further if play has multiple benefits, which in some species appears to be the case (Nahallage et al., 2016; Nunes & Monroy Montemayor, 2023). Different types of play may manifest different types of benefits, and perhaps “asking about the adaptive significance of play is rather like asking about the adaptive significance of ‘goal‐oriented’ behaviour” (Wolf, 1984, p.183). Play may also have radically different evolutionary origins than its current functional benefits today (Pellis et al., 2015), adding further impediments to a clear and simple unitary explanation.

Although play has now been documented across a diverse range of animal taxa (Burghardt, 2005), the majority of well‐known and studied cases are found in mammals (Fagen, 1981). Within playing mammals, although adult play is seen in some species, play is predominantly exhibited in juveniles. The fact that play is often so concentrated during youth raises many developmental questions regarding why play seems to be only an essential activity in younger individuals. Burghardt (2005) has suggested that play is widespread among young mammals because they are provided with a reliable source of energy and nutrients by the lactating mother. If parental provisioning is very important for the growing young, then the cessation of it (i.e., weaning in mammals) should profoundly affect the ontogenetic course of independent foraging, and hence time allocation to other types of behaviour. From this perspective, three periods of ontogeny could be distinguished: pre‐reproductive dependent, pre‐reproductive independent and reproductive. When modelling the ontogenetic course of play, these three periods should be taken into account. It has also been shown that different types of play appear at different times in ontogeny (e.g. Cuvier's gazelle, *Gazella cuvieri*, Gomendio, 1988; South American fur seal, *Arctocephalus australis*, Harcourt, 1991), and this may be due to these play forms having different functions within a species.

Perhaps due to its paradoxical nature, play has only received relatively recent attention from the modelling community, with the questions posed and methods used through theoretical analysis being diverse. Many of these approaches have focussed on various aspects of social play, looking at the possible beneficial consequences on later social behaviour (Cenni & Fawcett, 2018; Dugatkin & Bekoff, 2003; Durand & Schank, 2015; Grunloh & Mangel, 2015; Schank et al., 2018) and even structural features, such as strategies within play‐fighting (Bell et al., 2015). Elsewhere, it has been shown that different types and complexities of play (or no play at all) are likely to evolve in different taxonomic lineages according to the marginal energetic costs that the play imposes (Smaldino et al., 2019). Prior to these more recent approaches, Fagen explored various conditions for the evolution of play, through the equilibria of allele frequencies for innovation (Fagen, 1976b), or through assuming immediate costs and delayed benefits and showing the consequences on life‐history (Fagen, 1977). Elsewhere, it has been shown that frivolous play can emerge simply as a consequence of constrained energy resources in the population, where excess usage depletes resources that would benefit competitors, showing that direct functional benefits of play may not be entirely necessary (Auerbach et al., 2015).

In this paper, we model an organism choosing between the activities of foraging, resting, and playing. For this model, we assume that play can improve abilities of a certain ‘serious’ activity, but not as well as doing that activity itself. In this case, foraging is the best practice for foraging, with play being an inferior form of practice. Play, which is often exaggerated, boisterous and extravagant, may also have similar, if not greater energetic costs associated with it. The question then remains, why play when you can achieve better learning outcomes, spend less energy, and at the same time be productive towards one's survival and reproductive goals? In these circumstances, what can play offer?

One possibility is that, all things considered, play is a safer activity. Being ‘non‐serious’, play is void of serious consequences, and although that feature normally points to the lack of obvious benefits, it also allows for a reduction of risks and dangers that could lead to injury or death. Perhaps the most obvious case of this is with play‐fighting, where both participants restrain from causing actual harm to one another (Pellis & Pellis, 2017). Also, in contrast to foraging, play does not fulfil any urgent physiological needs, does not depend on food availability, and does not require travelling across exposed space between food sources. Because of this, the animal can allocate play to safer time–space windows. This is consistent with the idea that play occurs only when the animal is free from external stressors and threats (Burghardt, 2005; Held & Špinka, 2011; Loizos, 1967; Panksepp, 1998; Siviy et al., 2006), and appears to occur more often in domestic or captive animals than in the wild (Himmler et al., 2013). This echoes Huizinga's talk of play always proceeding “within its own proper boundaries of time and space” (1949, p.13), although the intensity of focus often found during playful bouts can often lead to a dangerous lack of vigilance (Harcourt, 1991) or even a significant exposure to pathogens (Kuehl et al., 2008). Nonetheless, for the model that follows, we assume that the predation rate during play is lower than in the hostile world encountered during foraging.

We focus on foraging behaviour (as the main ‘serious’ alternative to play) because energy is one of the fundamental resources necessary for survival and reproduction. The gain of energy through foraging therefore has a very clear causal relationship to reproductive value. It also allows us to capture some features of the energy costs of the alternative activities and combine them into a simpler common currency. We will explore three main versions of this model, each of which will assume that play is a practice for a different kind of ability. These will be *Play‐as‐improving‐antipredator‐ability*, *Play‐as‐improving‐foraging‐success‐ability*, and *Play‐as‐improving‐reproductive‐ability*. Each of these ability improvements may lead to improved performance on some aspect of behaviour, which may ultimately lead to improvements in final reproductive success. The structure of the model means that, with some minor adjustments, foraging (to increase energy reserves) could just as easily be substituted with another ‘serious’ adult behaviour (that increases some other ecologically relevant state variable), without loss of generality.

## 2 BASIC MODEL

The model considers a mammal who must forage for food, but also has the option to play or to rest. We utilise a state‐space approach to consider the value of different actions in the different states which the animal may find itself. An individual has energy reserves (*x*) and some ability (*a*). For each period of time (for each moment at *t*), the animal can choose which activity to engage in, each having different consequences on its future state. This should not be interpreted as the only activity in a given time period, but as an additional focus of activity after basic subsistence energy procurement, sleep and grooming needed to achieve homeostatic maintenance and survival. This assumed background activity is not explicitly modelled, and the choice behaviour in the model is focussed on what the animal does in its remaining ‘free time’. The additional chosen activity may contribute extra energy to put towards growth (additional foraging) or cost extra energy but have different consequences (e.g. play or rest). This abstraction is necessary in order to focus on the choice behaviours that relate to play, without having to model many other state variables and interactions that would greatly complicate the interpretation of the results.

Energy reserve levels must be kept above 0 or the individual dies of starvation. Let us define U*(*x*,*a*,*t*) as the value of an individual's life if it performs the optimal action for every choice it makes for its ‘free‐time’ behaviour. The individual is modelled for a total of *T* time‐steps (makes *T* choices), at which point there are no more choices left to make. If the individual has at least a certain critical amount of energy (*x*_crit_) at *T*, the individual is rewarded with a score of 1, whereas if this level of reserves is not attained by time *T*, the individual receives a score of 0. This type of terminal value function could be interpreted in numerous ways, depending on the life history of the species – for simplicity, we consider this to be sexual maturity, with *x*_crit_ representing the minimal energetic reserves for successful reproduction.

During the first part of its life (pre‐reproductive dependent, lasting until the ‘weaning’ time *t* = *τ*), the animal is a dependent offspring and it receives some energy provisions (e.g. milk, foraged prey provisions, etc.) from its parent (energy amount *w*_*e*_ with probability *w*_*z*_). In the second stage of its life (pre‐reproductive independent), the parent no longer provides, and the individual can only obtain extra energy from foraging. What we are modelling here is the time from which a new offspring is able to move freely on its own (*t* = 0) until their sexual maturity (*t* = *T*). The availability of parental provisions is the only difference between the two phases modelled here, with the pre‐reproductive dependent having a greater buffer from starvation, which may promote playful behaviour.

At every timestep, the animal has a choice between three different activities: it can forage, it can play, or it can rest. Foraging has energy costs (*c*_f_) and yields energy returns stochastically (amount *y*_*e*_ with probability *y*_*z*_). It also has a certain risk of predation associated with it (*m*_*f*_). Play has the same (or an even greater) energy cost as foraging (*c*_*p*_) but yields no energy gains in return. Play however has a lower risk of predation (*m*_*p*_), since it happens in a safe space, and, when young, supervised by an adult. Finally, individuals can also rest, and this entails the lowest energy costs (*c*_*r*_) and predation risk (*m*_*r*_), but there are no other consequences. The energy costs of the different activities are summarised as *c*_*r*_ < c_f_ ≤ *c*_*p*_ and mortality rates as *m*_*r*_ < *m*_*p*_ < *m*_*f*_.

In these models, both the activity of play and the activity of foraging also probabilistically increment the animal's ability (by probability *s*_*p*_ and *s*_*f*_, respectively). This improved ability has some beneficial impact for the animal, and these benefits are different for each model (in Model 1, it reduces predation risk whilst foraging, in Model 2, it increases foraging success rate, and in Model 3, it increases the probability of successful reproduction and thus directly improves the payoff at time *T*). The mechanics of the ability concept and how it affects performance of other activities will be discussed for each of the models in the specifications below.

The value of a given activity in a given state is defined by the function *U*_activity_(*x*,*a*,*t*). Using stochastic dynamic programming (Bellman, 1957; Houston & McNamara, 1999; Mangel & Clark, 1988), we can iterate backwards from *T*, to determine the value of being in each possible state in the model. From this, we can also determine the optimal choice for any given scenario that the individual might find itself in. The technical details of the model are shown in the Appendix A and the different default parameters of the model can be found in Table 1.

**TABLE 1:** Parameters in the model, their interpretation, and their default values.

Symbol	Interpretation	Default parameter values	Range of parameter where play is predicted for at least 5% of lifetime (other parameters held constant)			
			Model 1	Model 2	Model 3a	Model 3b
*X*	Maximum energy reserves	300	All values (150–600)	All values (150–600)	All values (150–600)	All values (150–600)
*A*	Maximum ability level	100	*A* ≤ 121	40 ≤ *A* ≤ 100	All values (10–250)	60 ≤ *A* ≤ 150
*T*	End time of model	200	175 ≤ *T*	200 ≤ *T*	All values (50–350)	150 ≤ *T* ≤ 300
*τ*	End time of parental provisioning	50	*τ* ≤ 80	10 ≤ τ ≤ 50	All values (1–100)	All values (1–100)
*w*_*e*_	Provisioned energy amount	3	*w*_*e*_ ≤ 5	1 ≤ *w*_*e*_ ≤ 3	All values (0–10)	All values (0–10)
*w*_*z*_	Provisioned energy probability	0.7	All values (0–1)	0.2 ≤ *w*_*z*_ ≤ 0.7	All values (0–1)	All values (0–1)
*y*_*e*_	Foraging energy amount	5	3 ≤ *y*_*e*_	5 ≤ *y*_*e*_	3 ≤ *y*_*e*_	4 ≤ *y*_*e*_
*y*_*z*_	Foraging energy probability	0.7 (**Model 2:** *y*_*z*_(a): *y*_*z*_(0) = 0.1, *y*_*z*_(A) = 0.9)	0.5 ≤ *y*_*z*_	*y*_*z*_(0) ≤ 0.1	0.4 ≤ *y*_*z*_	0.5 ≤ *y*_*z*_
c_f_	Energy cost of foraging	2	c_f_ ≤ 3	c_f_ = 2	c_f_ ≤ 3	c_f_ ≤ 3
*c*_*p*_	Energy cost of play	3	*c*_*p*_ ≤ 5	*c*_*p*_ ≤ 3	All values (1–14)	*c*_*p*_ ≤ 12
*c*_*r*_	Energy cost of rest	1	1 ≤ *c*_*r*_	1 ≤ *c*_*r*_	All values (−1 to 3)	*c*_*r*_ ≤ 2
*m*_*f*_	Predation risk of foraging	0.002 (**Model 1**: *m*_*f*_(*a*): *m*_*f*_(0) = 0.005, *m*_*f*_(A) = *m*_*p*_)	0.0012 ≤ *m*_*f*_(0)	0.0017 ≤ *m*_*f*_	*m*_*f*_ ≤ 0.06	0.0003 ≤ *m*_*f*_ ≤ 0.015
*m*_*p*_	Predation risk of play	0.0002	*m*_*p*_ ≤ 0.006	*m*_*p*_ ≤ 0.00015	All values (0–0.06)	*m*_*p*_ ≤ 0.001
*m*_*r*_	Predation risk of rest	0.0001	All values (0–0.06)	0.0001 ≤ *m*_*r*_	All values (0–0.06)	All values (0–0.06)
*s*_*f*_	Probability of improving ability from foraging	0.8 (**Model 3a**: *s*_*f*_ = 0)	0.5 ≤ *s*_*f*_	*s*_*f*_ ≤ 0.27 & 0.46 ≤ *s*_*f*_ ≤ 0.8	All values (0–1)	All values (0–1)
*s*_*p*_	Probability of improving ability from play	0.5	0.3 ≤ *s*_*p*_	0.5 ≤ *s*_*p*_	0.01 ≤ *s*_*p*_	0.05 ≤ *s*_*p*_
*x*_crit_	Critical level of energy for reproduction at time *T*	150	90 ≤ *x*_crit_	150 ≤ *x*_crit_	All values (1–265)	All values (1–265)
*q*	Exponent transformation on final reserves to value	0	All values (0–2)	All values (0–2)	All Values (0–2)	*q* ≤ 0.05
*ω*_*z*_(*a*)	Probability of final reproduction	**Model 3a & 3b:** *ω*_*z*_(0) = 0.1, *ω*_*z*_(*A*) = 0.9	N/A	N/A	*ω*_*z*_(0) ≤ 0.75	*ω*_*z*_(0) ≤ 0.85
*x*_start_	Starting energy for Forward Model	50	*x*_start_ ≤ 160	30 ≤ *x*_start_ ≤ 100 & 280 ≤ *x*_start_	All values (1–280)	All values (1–280)
*a*_start_	Starting ability for Forward Model	0	*a*_start_ ≤ 85	*a*_start_ = 0	*a*_start_ ≤ 90	*a*_start_ ≤ 40

*Note*: The final four columns show the range of values for each model over which the proportion of predicted lifetime activity of playing is at least 5% (holding all other parameters constant). This was done through a one‐at‐a‐time sensitivity analysis, by using the parameter values of each basic model, and varying the parameter in each row. For each parameter value variation, we would run the forward model to predict the amount of time individuals spent doing each activity over the total time modelled (*t* = 0 to *T*). The default parameters are usually the same for every model, although for each model variation, one of these is a variable that is a function of ability (shown in bold). How each activity changes with the varying parameter can be seen in the figures in the Appendix (Figures A1, A2, A3, A4), and explanations of those of interest are also given.

The result of the dynamic programming procedure produces a lookup table of the value of each possible choice (*U*_forage_, *U*_play_, *U*_rest_), the greatest of which is the optimal value (the expected value of being in a specific state, assuming all future choices are optimal, U*(*x*,*a*,*t*)). The optimal choice for a specific state, *Ψ*(*x*,*a*,*t*), a nominal output variable, is just the activity which has the optimal value. However, this output does not say which states an individual is likely to find themselves in. By using the choice function and selecting some initial conditions (*x* = *x*_start_ and *a* = *a*_start_) we can run the model forward to see the probability that a given individual (or equivalently, the proportion of individuals in a population), acting optimally, will end up in each of the future states. From this, we can also calculate the total proportion of individuals that would be found doing a given activity with their excess time at any given timestep. Together, the optimal choices and the expected lifetime activity budgets predicted by the model can offer some insight into the effects on behaviour (and the reasons for those effects) that different environmental conditions might have. See the Appendix A for all the technical details of each calculation for these models.

**FIGURE A1:**
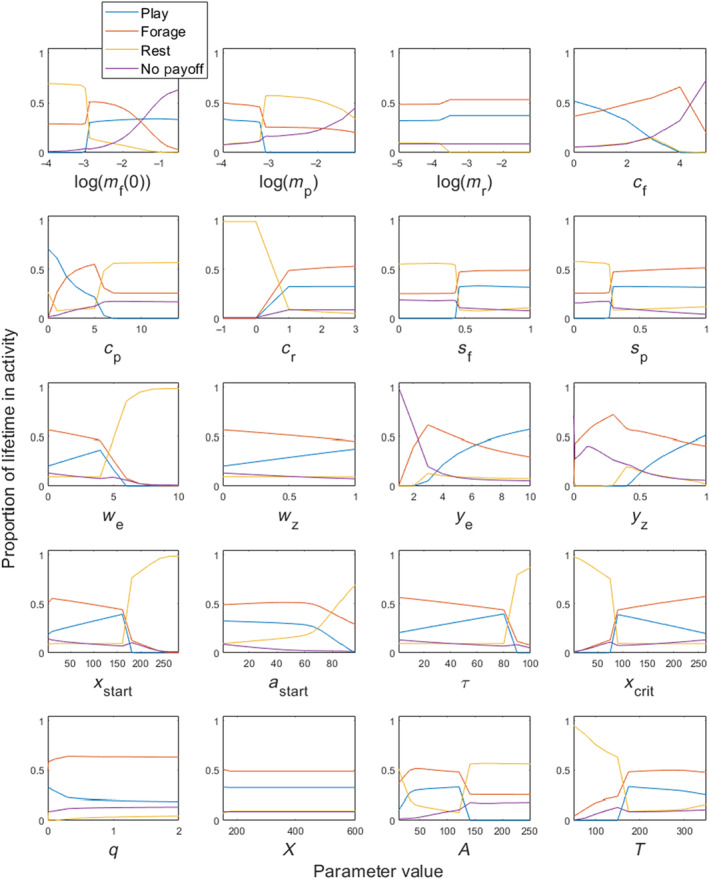
Sensitivity analysis for each parameter for Model 1: Play as increasing Predator Avoidance. Effects of modifying one parameter on the proportion of time spent in each activity over the total time of the model. When modifying one parameter, all other parameters are held constant as the default value (see Table 1, main text). Blue = Play, Red = Forage, Yellow = Rest. Purple = Proportion of individuals dead or in a state where it is impossible to attain *x*_crit_ (and therefore receive a payoff) on average across the total time of the model.

**FIGURE A2:**
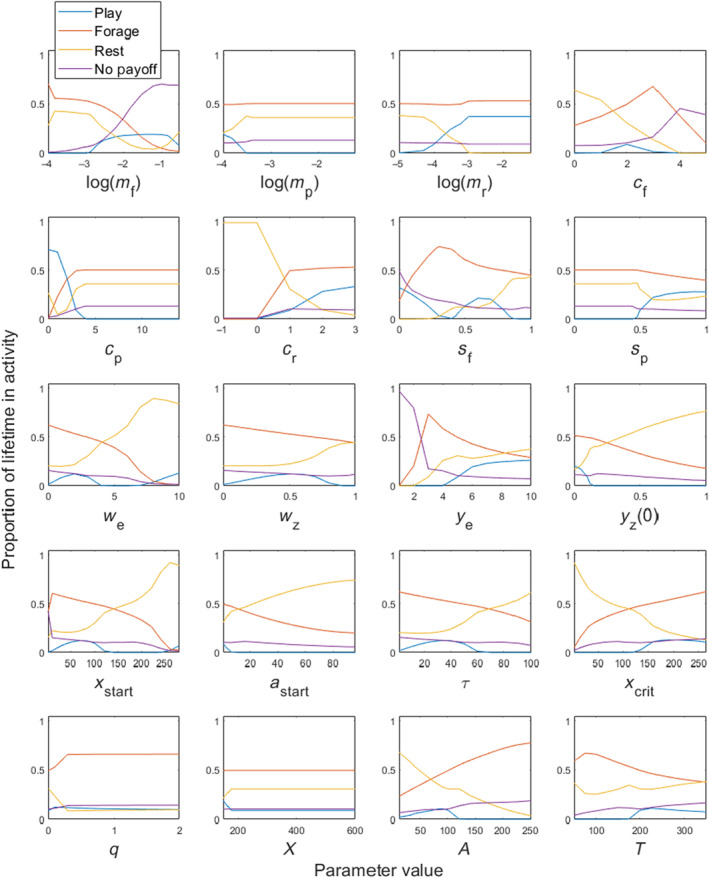
Sensitivity analysis for each parameter for Model 2: Play as increasing Foraging Success. Effects of modifying one parameter on the proportion of time spent in each activity over the total time of the model. When modifying one parameter, all other parameters are held constant as the default value (see Table 1, main text). Blue = Play, Red = Forage, Yellow = Rest. Purple = Proportion of individuals dead or in a state where it is impossible to attain *x*_crit_ (and therefore receive a payoff) on average across the total time of the model.

**FIGURE A3:**
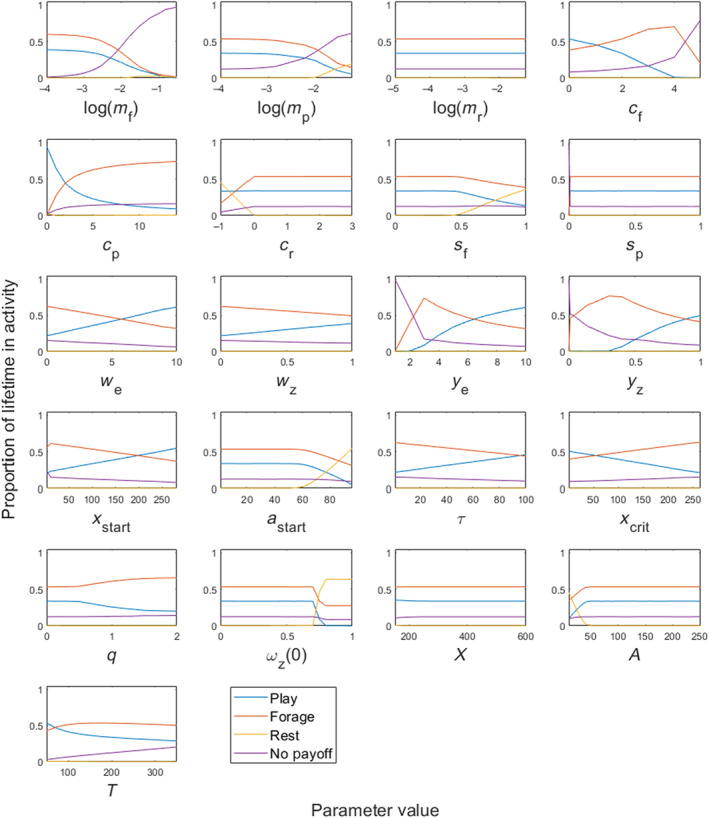
Sensitivity analysis for each parameter for Model 3a: Play as increasing Reproductive Success, *s*_*f*_ = 0. Effects of modifying one parameter on the proportion of time spent in each activity over the total time of the model. When modifying one parameter, all other parameters are held constant as the default value (see Table 1, main text). Blue = Play, Red = Forage, Yellow = Rest. Purple = Proportion of individuals dead or in a state where it is impossible to attain *x*_crit_ (and therefore receive a payoff) on average across the total time of the model.

**FIGURE A4:**
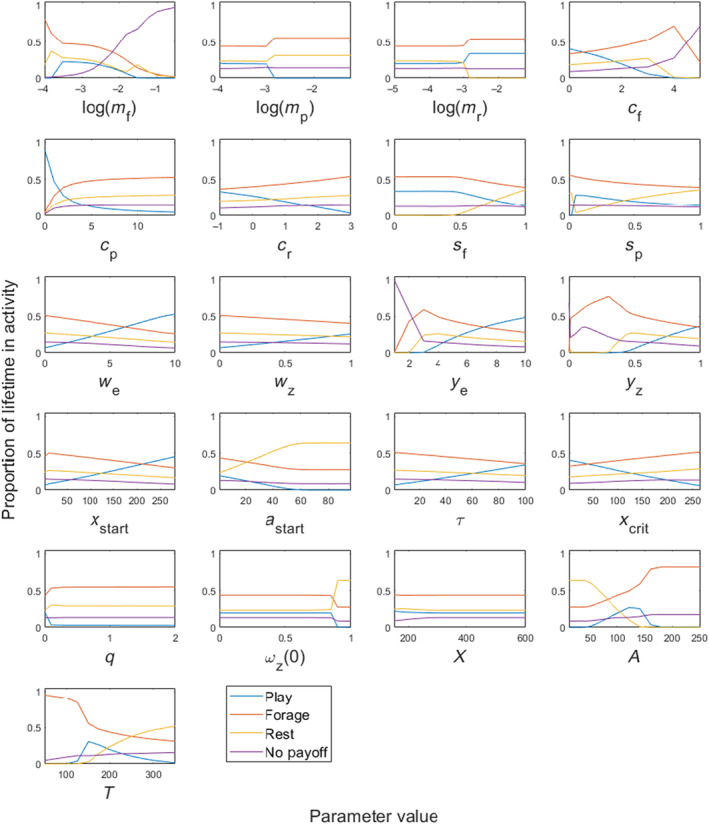
Sensitivity analysis for each parameter for Model 3b: Play as increasing Reproductive Success, *s*_*f*_ = 0.8. Effects of modifying one parameter on the proportion of time spent in each activity over the total time of the model. When modifying one parameter, all other parameters are held constant as the default value (see Table 1, main text). Blue = Play, Red = Forage, Yellow = Rest. Purple = Proportion of individuals dead or in a state where it is impossible to attain *x*_crit_ (and therefore receive a payoff) on average across the total time of the model.

We can also perform a sensitivity analysis on each of these models. This can be done by modifying one parameter at a time over a range of values whilst holding all the other parameters constant. For each parameter setting, an optimal choice function can be generated using dynamic programming, and then with a choice of initial conditions, the model can be run forward, and the proportion of excess time spent doing each activity over the entire lifetime can be calculated by summing the probabilities of being in each state for each optimal activity. A crude summary of the sensitivity analysis can be seen in Table 1 (showing the range of values where at least 5% of the lifetime free choices are for play behaviour). More detailed results of the sensitivity analysis are shown in the Appendix (Figures A1, A2, A3, A4).

The models that follow will be distinct versions of the base model described above, utilising the same default parameters, and differing only in the consequences of being in a state of increased ability. The type of ability that play improves would likely vary across taxa, which encounter different ecological problems. We realise that the likely species that each model applies to may vary in their life‐history substantially and the models may need modifying to capture relevant features. However, we have kept the models as similar as possible to maximise model comparability, allowing the differences in the patterns of play behaviour to be attributable to differences in ability type. The simplifications in the variables and their interactions made in the dynamic programming approach mean that it will be difficult to make quantitative predictions, rather it should be expected to demonstrate only qualitative predictions about where play might be found during development.

### 2.1 Model 1: play as improving antipredator ability

For the first instantiation of the model, we consider a case where play is an activity that offers improvements in an individual's antipredator abilities. Many prey species are relatively vulnerable to predation whilst foraging for food, and various forms of locomotor‐rotational play may improve general movement abilities or specific skills that could mitigate the chances of being predated, including the individual's vigilance, reaction time, speed and agility of escape behaviour, and the ability to fight off an attacker. More importantly, the activity of play need only improve one of these skills to have a significant impact on the individual's mortality risk. This model is most representative of some kind of grazing ungulate, a taxon known for its locomotor play (e.g., Gomendio, 1988).

In this first model, the predation risk associated with foraging is a function of an individual's ability (*m*_*f*_(*a*)). This predation risk decreases linearly with increasing antipredator ability, such that *m*_*p*_ = *m*_*f*_(*A*) << *m*_*f*_(0), that is, there is a high mortality whilst foraging when ability is low (*a* = 0) and a low mortality (the same as play) when foraging ability is high (*a* = *A*). By foraging, the individual's antipredator abilities become improved with each foraging session (improvement of 1 with probability *s*_*f*_). Like foraging, play also improves antipredator abilities (improvement of 1 with probability *s*_*p*_), but it improves them at a lower rate than real life foraging – i.e. in this model, the best practice for safe foraging is foraging (*s*_*f*_ > *s*_*p*_). The detailed formula and computations specific to each model can be found in the Appendix A.

### 2.2 Results for Model 1

**FIGURE 1:**
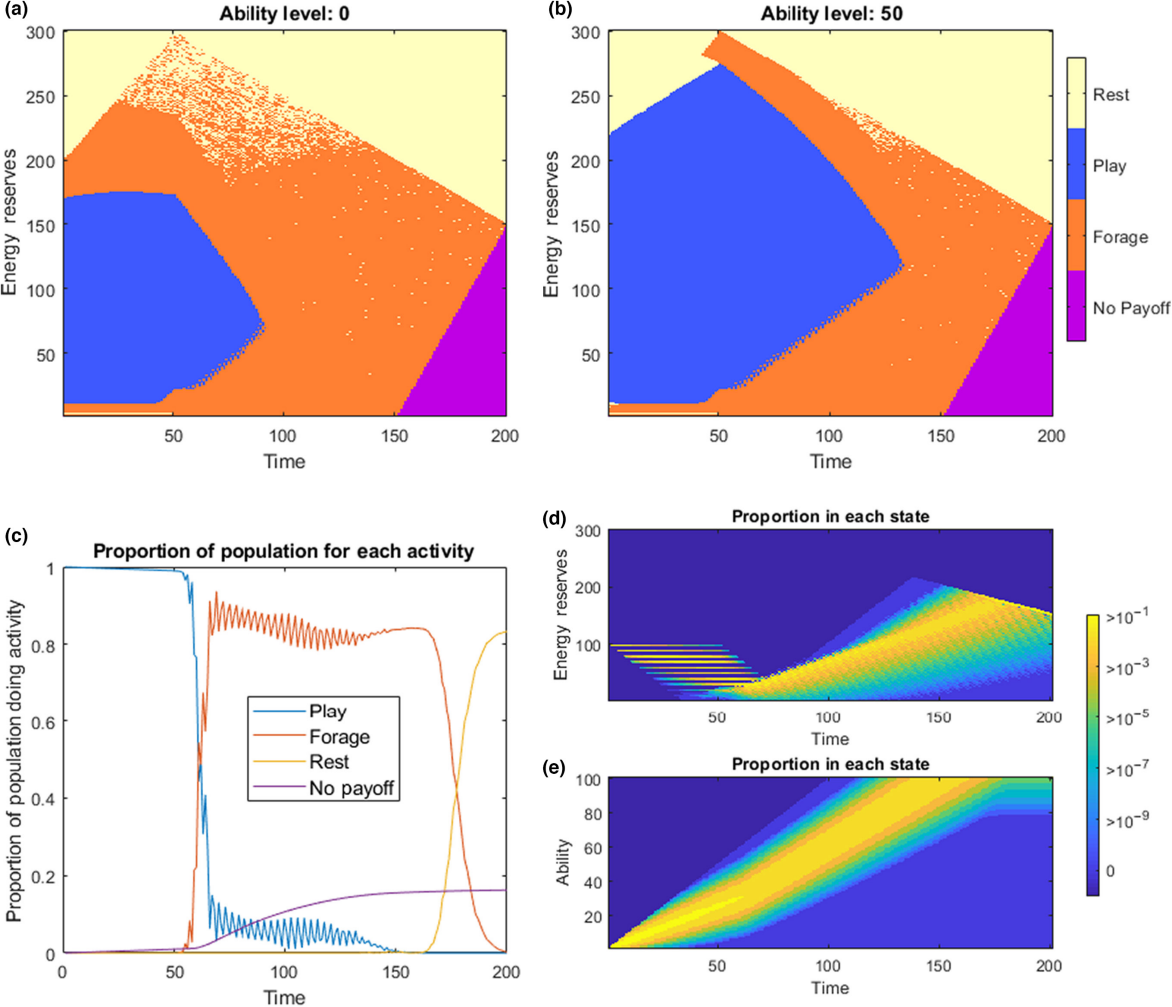
The *Play as improving Antipredator Ability* model. The top panels show the choice function determined by stochastic dynamic programming for the default parameters. (a) shows the optimal choice in regions of state space across time and energy reserves for *a* = 0. (b) shows the optimal choice in regions of state space across time and energy reserves when ability is half the maximum value (*a* = 50). The purple area is a region where the individual is unable to reach the critical level of energy reserves for a final payoff. (c) shows the proportion of optimally behaving individuals choosing each activity at any given time (from the forward model with initial conditions of *x*_start_ = 100 and *a*_start_ = 0). The ‘No Payoff’ individuals here include those that have died (from predation or starvation), and those who cannot reach *x*_crit_ before time *T*. The bottom right panels show the proportion of individuals ending up with different energetic levels, (d), and ability levels, (e), over time for a population starting at initial conditions *x*_start_ = 100 and *a*_start_ = 0. The bright yellow states are where at least 10% of an optimally behaving population would be found, and blue colours being where fewer than 1 in a million optimally behaving individuals would end up.

The first key output of the model is the optimal choice function for each three of the state variables, *Ψ*(*x*,*a*,*t*), and this can be plotted onto a two dimensional space when holding one of the state variables constant (e.g. optimal choice at a given time and level of energy reserves, for a certain level of ability). This can be seen in Figure 1a,b, for *a* = 0 and for *a* = 50. We can see that play is optimal at most intermediate levels of reserves whilst the individual is still receiving energetic provisioning from a parent, and so starvation is not a serious threat (at least compared to being predated whilst foraging when having a low ability). At very high levels of reserves, either the individual can reach the critical threshold (*x*_crit_) for the remaining time just by safely resting, or the individual may only have to do additional foraging for growth a few times in its life in order to achieve *x*_crit_ by time *T*, and therefore the reduced mortality associated with play is not worth the extra foraging that would be needed to compensate for the energy spent in play. At very low reserves, the individual should forage. This is because the energetic provisioning is probabilistic, and so there is a reasonable risk of starvation if several consecutive time‐steps yield no energy, and therefore the higher mortality associated with foraging with low ability must be faced in order to mitigate the risk of starvation. Once the animal is weaned of the parental provisioning at time *t* = *τ* = 50, the window of energy reserves where play is optimal decreases, although it stretches to a later time in individuals who have already obtained a higher ability (Figure 1b). Although very little play can be found after the halfway point to sexual maturity if current ability is low, when abilities are higher, play can still be found at *t* = 160. At very low energy reserves, or in the second half of the period, individuals should forage, since individuals need to reach the critical threshold to obtain any payoff. The purple triangle at the bottom right hand corner of Figure 1a,b is an area of state space where the individual could not possibly reach *x*_crit_ by time *T*, and so no behaviour is optimal and no final payoff can be achieved.

If we run the model forward, starting with initial conditions of *x*_start_ = 100 and *a*_start_ = 0, we can see the proportion of individuals doing each activity, from a population acting optimally across the time period (Figure 1c). The purple line shows individuals who cannot achieve a payoff in the model. This is the cumulative count of those individuals who reached a state of starvation (*x* = 0) or were unfortunate enough to be predated during their choice of activity, and after *t* = 150, those who cannot possibly reach *x*_crit_ by time *T*. For the first 50 time‐steps, play is the only activity found in the animals' free choice behaviours, whereafter it drops off steeply, being maintained at lower rates in the population until around *t* = 150. Between *t* = 100 and *t* = 150, obtained abilities are already quite high, which further extends the range of values for which play is optimal to even later in life.

Similarly, we can plot the density of individuals in terms of their state, for both energy reserves (Figure 1d) and for ability (Figure 1e). The bright yellow areas are where the highest proportion of individuals are found (at least 10% of individuals for any given state: *x*,*a*,*t*), with proportions falling off by an order of magnitude with each change in shade of colour. From the initial conditions, the majority of individuals play whilst being provisioned by the parents. From there, there is a large shift to foraging behaviour, until near the end of the model, where the individual is on a trajectory of enough reserves to remain above *x*_crit_, after which rest is the main free‐time activity for the remainder of timesteps until *T*. Most individuals reach their maximum ability level (*a* = 100) by *t* = 150 (Figure 1e). Most of these overall ability improvements come from actual foraging itself, although all the ability improvements during the dependent period come from play.

A one‐at‐a‐time sensitivity analysis shows a wide range of parameter values over which we predict play for at least 5% of lifetime activity choices (whilst holding other parameters constant). These ranges are shown for each model in this paper in Table 1 and are shown in more detail graphically and discussed further in the Appendix (Figure A1). One key finding from this is that the lowest value for the initial foraging mortality probability (*m*_*f*_(0)) is 0.0012, which is around six times the probability of mortality from play. This is a quite significant but not altogether implausible difference.

Skills that assist in predator avoidance are just one of many possible instantiations of the ability concept, and with some minor adjustments to this model, we can also explore the potential for play to improve abilities in other ‘serious behaviours’, firstly in foraging success, and secondly in reproductive behaviour.

### 2.3 Model 2: play as improving foraging success ability

For the second model, we again consider an animal playing to improve ability, where this time ability affects an individual's foraging success. This may be most applicable to a predatory species, where finding, stalking, pouncing, catching, and handling mobile prey is necessary for the animal to acquire their food, and being a competent predator is the difference between a successful hunt and complete failure. This model is most representative of some kind of predatory feline, a taxon known for its object play (e.g., Bateson et al., 1990).

For this model, we set the probability of foraging success as a linearly increasing function of ability, *y*_*z*_(*a*), such that 0 ≤ *y*_*z*_(0) << *y*_*z*_(*A*) ≤ 1. As in the first model, both the activity of foraging and the activity of play increases the ability state variable probabilistically (increment of 1 with probability *s*_*f*_ and *s*_*p*_ respectively, where *s*_*p*_ < *s*_*f*_), and as before, the best practice for foraging is foraging, with play being an inferior way to improve ability. The impact of ability on mortality when foraging is removed such that *m*_*f*_ is now a constant and unaffected by foraging or playing experience. The remainder of the details of the model are the same (see the Appendix A for the modified calculations). Here, we have used the default parameters of *y*_*z*_(0) = 0.1 and *y*_*z*_(*A*) = 0.9, suggesting that foraging performance can be radically improved through either form of practice.

### 2.4 Results for Model 2

**FIGURE 2:**
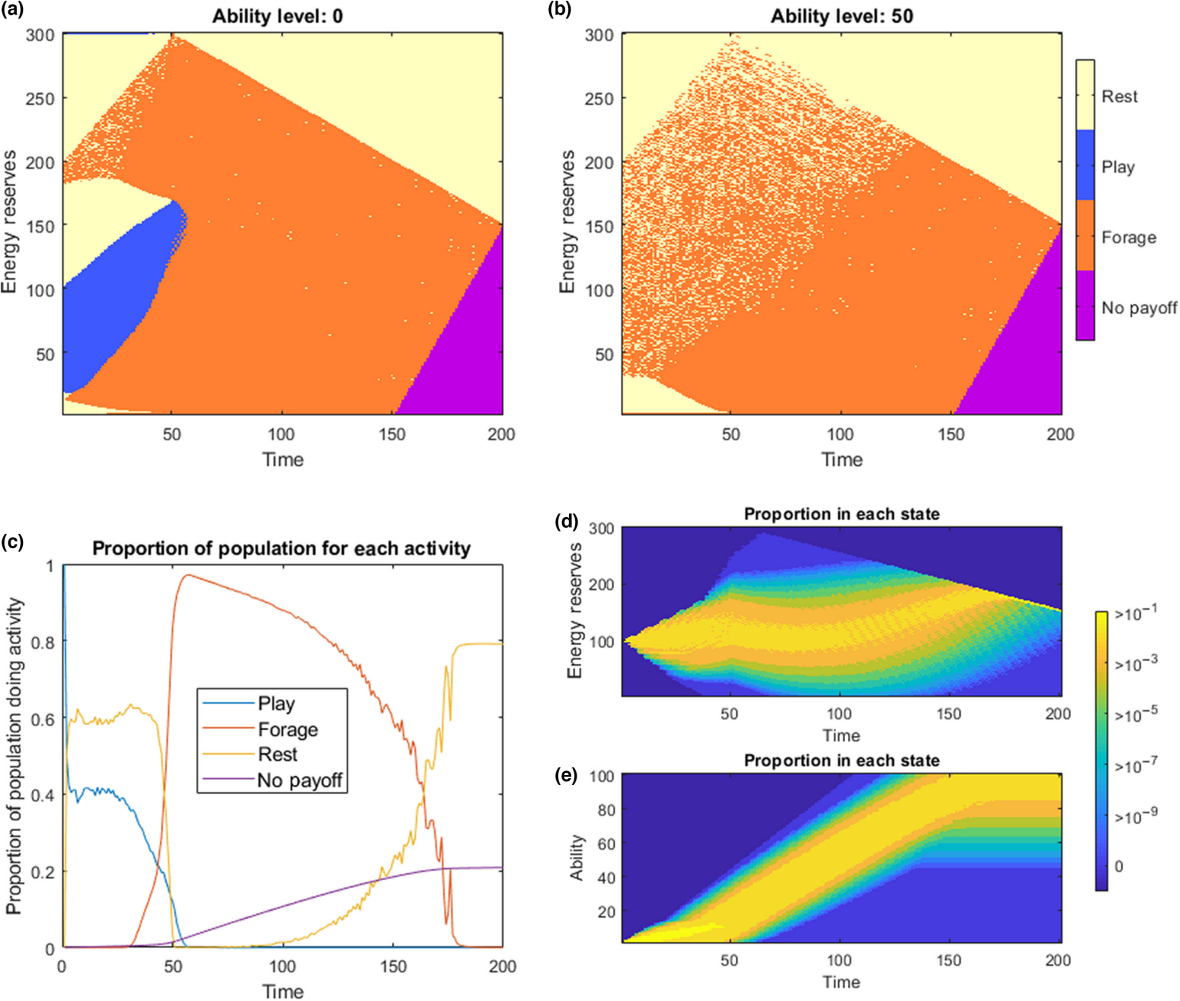
Results of the *Play as improving Foraging Success* Ability model. (a) and (b) show the optimal choices in regions of state space across time and energy reserves for *a* = 0 and for *a* = 50 respectively. The purple area is a region where the individual is unable to reach the critical level of energy reserves for a final payoff. (c) shows the proportion of optimally behaving individuals choosing each activity at any given time (from the forward model with initial conditions of *x*_start_ = 100 and *a*_start_ = 0). The ‘No Payoff’ individuals here include those that have died (from predation or starvation), and those who cannot reach *x*_crit_ before time *T*. The bottom right panels show the distribution of individuals ending up with different energetic levels, (d), and ability levels over time, (e), for a population starting at initial conditions *x*_start_ = 100 and *a*_start_ = 0. The bright yellow states are where at least 10% of an optimally behaving population would be found, and blue colours being where fewer than 1 in a million optimally behaving individuals would end up.

The choice function output of the model shows that play is optimal during the very early stages of life, at low to intermediate levels of reserves (Figure 2a). This range of reserves where play is the optimal choice rapidly narrows as time goes on, and play is essentially never optimal after parental provisioning ends (*t* = *τ* = 50). Play is also never an optimal strategy once a certain level of ability is already attained (Figure 2b), whereafter all ability improvements happen through foraging.

When the model is run forward with initial conditions of *x*_start_ = 100 and *a*_start_ = 0, we see that play occurs for 40% of the population at any one time for the first 30 timesteps, before falling to zero rapidly at the weaning time (Figure 2c). The patterns of energy reserve growth (2d) and ability improvements (2e) are very similar to those seen in Model 1. As with Model 1, the range of working parameter values can be seen in Table 1 and a more detailed sensitivity analysis is also included in the Appendix (Figure A2). One thing to note from this is that play in Model 2 is critically dependent on receiving sufficient parental provisions (*w*_*e*_, *w*_*z*_) and for a sufficient period of their early life (*τ*). This is in contrast to the other model instantiations, where the effects are not entirely dependent on any provisioning (although they are certainly enhanced by it). It is also important to point out that play only occurs when the initial foraging success rate is sufficiently low (*y*_*z*_(0) ≤ 0.1), that is, the individual must start their life being very bad at the task of foraging (catching prey).

### 2.5 Model 3: play as improving reproductive ability

The third instantiation of the ability concept we consider involves ability directly affecting the final payoff, by increasing the probability of successful reproduction at time *T*. Reproduction in many social mammal species requires a variety of social skills to find, attract, and copulate with a member of the opposite sex, and often for males, fighting with other males over access to females. It is plausible that various forms of social play could provide improvements in an individual's general or specific social abilities needed for these tasks, thus affecting their probability of sexual reproduction at maturity. We will call this the *Play‐as‐improving‐Reproductive‐Ability* model, and the full details of the modifications from the other models are specified in the Appendix A.

For this model, as before, we treat *T* as the age of sexual maturity, and the final payoff of as a function of both energy reserves and ability, making it necessary to pursue both ‘resources’ to achieve the maximum payoff. We set the probability of successful reproduction to be a linearly increasing function of ability (*ω*_*z*_(a)). We consider two almost identical versions of this model (differing by only a single parameter value) that leads to two very different interpretations.

For Model 3a, we again assume that play improves ability, but we remove any ability improvements generated through foraging (*s*_*f*_ = 0), as foraging does not intuitively offer practice for any of the skills needed for reproductive behaviour. This would be true for many social mammals, that live in closely knit groups, but obtain food through individual activity, such as various primate species or murid rodents, both of which are known for their social play (e.g., Pellis et al., 2023). In this case, the only way to improve ability is through play. For Model 3b, we assume that both play and foraging can improve ability, and, as in Model 1 and 2, that foraging offers even better social practice for reproduction than play (*s*_*f*_ > *s*_*p*_). This could be true for many social mammals who hunt cooperatively, such as many canines or cetaceans, both of which are also known for their social play (e.g., Cordoni & Palagi, 2019).

### 2.6 Results for Model 3a

**FIGURE 3:**
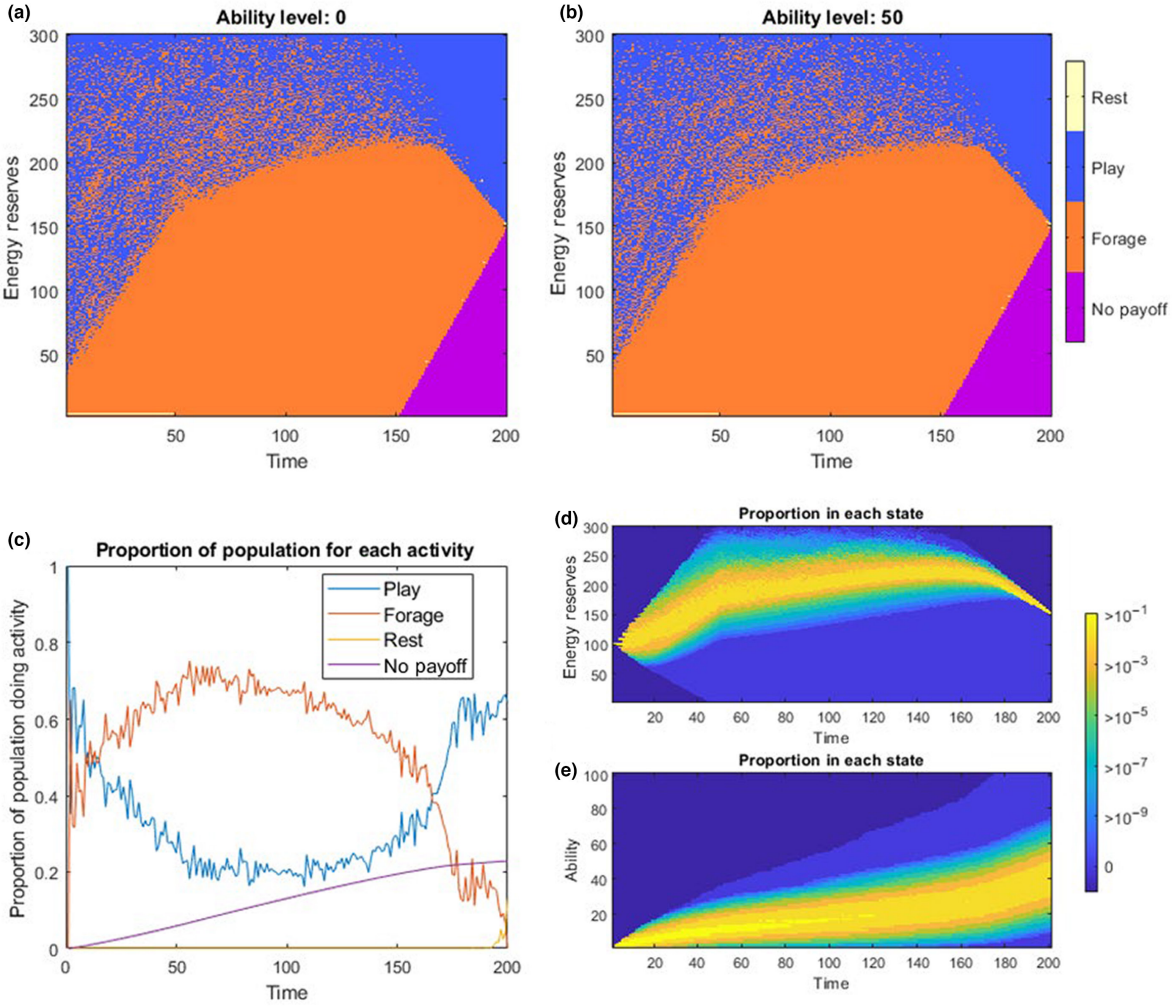
Results of the *Play‐as‐improving‐Reproductive‐Ability* model, where foraging does not increase ability (*s*_*f*_ = 0). (a) and (b) show the optimal choices in regions of state space across time and energy reserves for *a* = 0 and *a* = 50 respectively. The purple area is a region where the individual is unable to reach the critical level of energy reserves for a final payoff. (c) shows the proportion of optimally behaving individuals choosing each activity at any given time (from the forward model with initial conditions of *x*_start_ = 100 and *a*_start_ = 0). The ‘No Payoff’ individuals here include those that have died (from predation or starvation), and those who cannot reach *x*_crit_ before time *T*. The bottom right panels show the distribution of individuals ending up with different energetic levels, 3(d), and ability levels over time, 3(e), for a population starting at initial conditions *x*_start_ = 100 and *a*_start_ = 0. The bright yellow states are where at least 10% of an optimally behaving population would be found, and blue colours being where fewer than 1 in a million optimally behaving individuals would end up.

The output of the model shows that at low to medium reserves, individuals should focus their time on foraging to gain energetic reserves, and this is independent of their current ability level (Figure 3a,b). Individuals should also play throughout their pre‐reproductive life in order to improve their chances of final reproduction at sexual maturity, although this is normally done at higher reserve levels. In addition to this, individuals should almost never rest, since both foraging and ability feed into the final payoff function. The activity of play in this model has direct consequences on the final value (probability of successful reproduction). There are no reasons why play should happen earlier rather than later in development, since the benefits cannot be realised until the end of the model (at time *T*). This contrasts with the first two models, where earlier play has immediate tangible benefit (either through reducing predation risk whilst foraging (1) or increasing foraging success (2)) and these are instrumental to the necessary foraging behaviour that the individual must perform at some point to reach the critical threshold at time *T*.

When the model is run forward with initial conditions of *x*_start_ = 100 and *a*_start_ = 0, we see that play occurs in at least 20% of individuals during all timesteps, with an increase to around 60% of individuals as they approach sexual maturity (Figure 3c). The pattern of energetic reserve and ability increase also differs from the first two models, with a focus on growing energetic reserves much earlier in the model (since ability does not make foraging safer or more productive). After this, the average energetic reserves plateaus as foraging activity slowly decreases and playing activity increases (Figure 3d). Even though playing is chosen more in this model than the others, by sexual maturity, less than half of the individuals have achieved an ability level of half the maximum (at *T*, *a* ≈ *A*/2, Figure 3e) since playing is the only vehicle for improvements in ability (*s*_*f*_ = 0). Since there is always room for increasing the final payoff function by improving ability through play, individuals should almost never rest (except those precisely preserving enough energy close to *x*_crit_).

From the sensitivity analysis, we find that play is found, even when the conditions for play are particularly costly, such as when the probability of ability improvements from play are very low indeed (*s*_*p*_ = 0.01) and when the energy cost of play is unreasonably high (*c*_*p*_ = 14). Moreover, even when the difference between the reproductive probability at zero ability and at maximum ability is quite small (ω_z_(0) = 0.75 & ω_z_(A) = 0.9), and thus the benefits of improved ability are relatively small, significant amounts of play is still found. The robustness of play in this model derives from the fact that foraging cannot improve ability, and therefore play offers some unique benefits not found in the other activities.

### 2.7 Results for Model 3b

**FIGURE 4:**
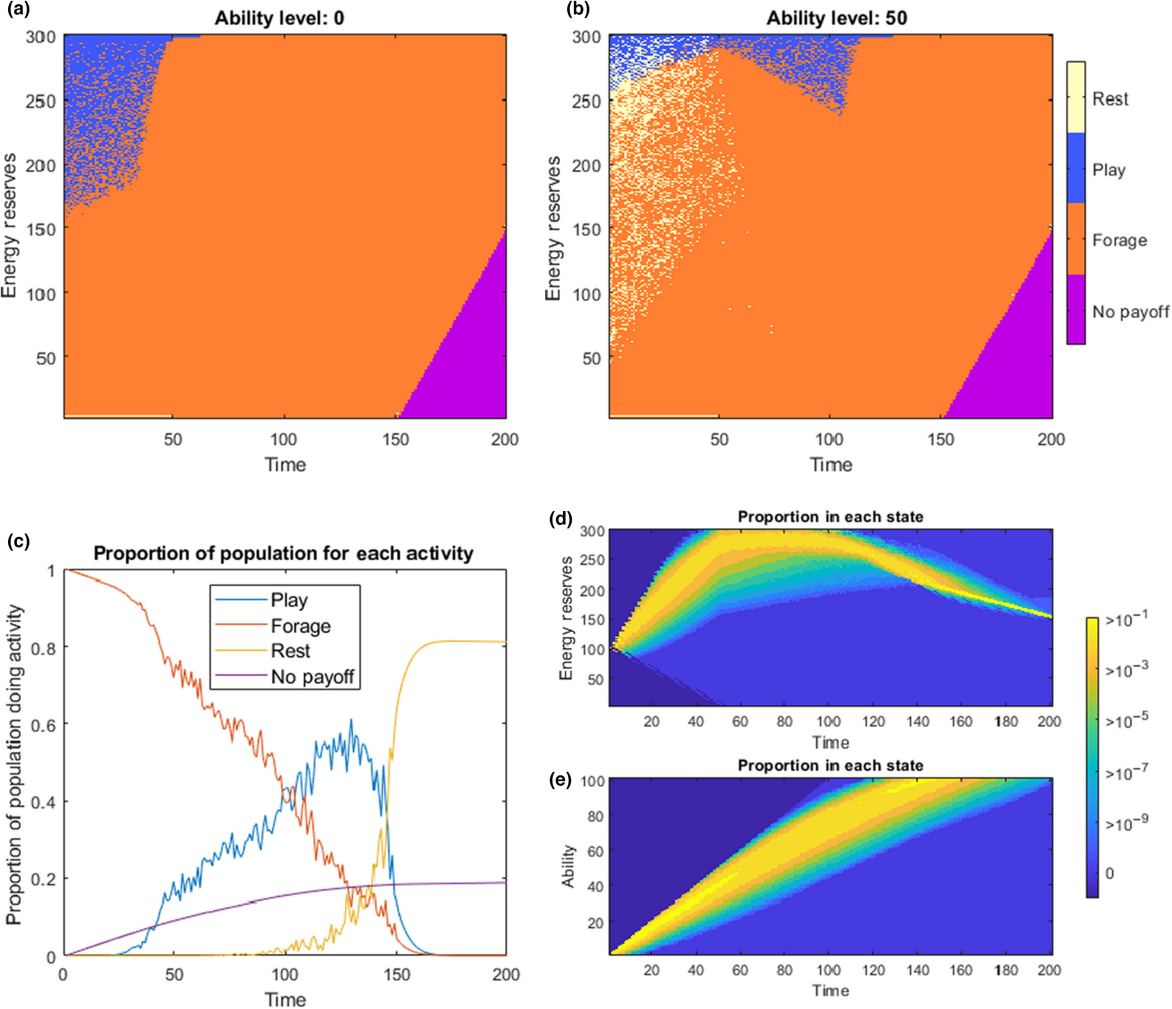
Results of the *Play‐as‐improving‐Reproductive‐Ability* model, where foraging also offers practice (*s*_*f*_ > *s*_*p*_). (a, b) The optimal choices in regions of state space across time and energy reserves for *a* = 0 and *a* = 50 respectively. (c) The proportion of optimally behaving individuals choosing each activity at any given time (from the forward model with initial conditions of *x*_start_ = 100 and *a*_start_ = 0). The ‘No Payoff’ individuals here include those that have died (from predation or starvation), and those who cannot reach *x*_crit_ before time *T*. The bottom right panels show the distribution of individuals ending up with different energetic levels, (d), and ability levels over time, (e), for a population starting at initial conditions *x*_start_ = 100 and *a*_start_ = 0. The bright yellow states are where at least 10% of an optimally behaving population would be found, and blue colours being where fewer than 1 in a million optimally behaving individuals would end up.

The output of the model shows that play behaviour only seems to be optimal at high levels of energy reserves (Figure 4a). As ability improves (which happens mostly through foraging at the beginning of the model), the range of states where play is optimal increases (Figure 4b). At higher abilities (*a* > 70), play is optimal for most states of high energy reserves (not shown in the figures here), as the latter increases in ability can be achieved more safely (*m*_*p*_ < *m*_*f*_, though less efficiently, *s*_*p*_ < *s*_*f*_) through play, and maximum ability (*A*) can still be attained before sexual maturity. When the model is run forward with initial conditions of *x*_start_ = 100 and *a*_start_ = 0, we see that individuals begin by foraging, and only start to play at *t* = 25 (Figure 4c). After this, the proportion of individuals playing increases and foraging decreases steadily until around *t* = 150, after which rest becomes the exclusive activity for the remaining 50 timesteps (when *a* = *A*, rest is optimal for most *x* > *x*_crit_, not shown in figures here). The death rate drops drastically halfway through the model, as most activity is shifted from the more dangerous foraging, to the safer activities of play and rest. Energy reserves reach their maximum levels (*x* ≈ *X*) after the end of parental provisioning at *t* = 50 (Figure 4d), and so some of the benefits of play observed may naively appear to be because the energetic benefits of foraging are absent when energy is at or near its ceiling. However, increasing the maximum possible energetic reserves (*X*) has no impact on the amount of play observed in the model results (see Sensitivity Analysis in the Appendix A). For most individuals, maximum ability is attained at around *t* = 140 (Figure 4e).

As with Model 3a, play is still found at significant levels, even when the probability of increasing ability through play is very small (*s*_*p*_ = 0.05) and when the energy cost of play is very high *(c*_*p*_ = 12). Since almost all surviving individuals reach maximum ability by time *T*, it may be worthwhile to achieve this as much as possible through play, due to its reduced mortality risks. A full sensitivity analysis for both of these versions of Model 3 can be seen in the Appendix (Figures A3 and A4).

## 3 DISCUSSION

The evolutionary benefits of play are unclear and hotly debated, and by most popular definitions, is characterised by the absence of immediately obvious function or purpose. In this modelling approach, we explored the interplay between three possible activities. At one extreme, rest is energy and predation risk minimising, but does not generate any improvements across any of the individual's states, and still causes a slow decline in energetic reserves. At the other extreme, foraging improves the individual's current energy reserves, as well as their instrumental ability variable, but with greater expenses in terms of energy cost (particularly if foraging is unsuccessful) and predation risk. Finally, there is the opportunity to play, which is not absolutely better than either rest or foraging for any of the costs or benefits but provides a safer place where its skills and abilities can be improved. The models presented here are very simple, abstracting away many features of play that may be relevant to its value in the real world. Adding additional features, costs, and possible activities could add more realism to the model at the expense of difficulty of interpreting the results. We have not assumed anything about the form of play, but kept play as a simple activity, offering potential improvements in ability for the alternative (serious) activity. Play could arbitrarily be substituted with other kind of directed practice, training, or general learning throughout this paper, raising further questions about why play exists as an activity (see next section).

As noted widely across the literature, play appears to be a highly heterogenous class of behaviour, creating difficulties for basic prescientific tasks such as definition, categorisation, and measurement (Burghardt, 2010; Smith et al., 1985). Our model merely supports the idea that a seemingly inconsequential activity, with higher energy costs and a lower learning potential than the activities which it emulates, could be functional in youth due to its lower‐risk character. Our model says very little about the origins of play, but instead provides a possible explanation for its current function and persistence as a behaviour across a wide range of taxa. Earlier in evolutionary history, play could have emerged in populations with no initial benefits, with the plausible advantages acquired only later, stabilising play as an adaptive behaviour (Burghardt, 2005; Pellis et al., 2015). In some lineages, play may have been present in earlier ancestors, only to be lost by some of the descendants (Pellis & Iwaniuk, 1999), and, for cases where play offers practice benefits, our model may also be useful in predicting conditions where such traits could be lost.

Our modelling approach shows some of the conditions where we might expect to find play behaviour. The results show that play may be adaptive under a wide range of conditions (Table 1) and that at a certain time during early ontogeny, play may be present in a majority (Figures 1c, 3c) or at least in a sizeable proportion of individuals (Figures 2c, 4c). This is in agreement with the fact that in juvenile mammals, play is usually exhibited by a majority, if not all the individuals of a species (Bertelsen & Jensen, 2019; Brown et al., 2015). However, the predictions of play behaviour in our model depends on the mortality risks of play being substantially lower than the mortality risks associated with foraging, and this may not be the case in all species. Our model contains many abstractions, with many important features not modelled explicitly, and as with most dynamic programming approaches to modelling, the predictions are generally only qualitative, showing the direction of change when features of the model are modified, and exposing the logic behind adaptive behaviour. As predicted, the cessation of parental provisioning (at time *τ*) causes an important shift in time allocation in Models 1 and 2. In these Models, the probability of play decreases swiftly after weaning. (Figures 1a–c, 2a–c). In Models 3a and 3b, weaning time also marks changes in behavioural development, yet these are more difficult to interpret.

These models make a number of other general predictions regarding the ontogeny of play according to what abilities the type of play actually offers practice for. The different courses of play in the models are in accordance with empirical findings that different types of play often peak at different ages (Gomendio, 1988). In Models 1 and 2, play improves ability for an intermediate activity (foraging), that must itself be performed in order to attain an energy target to succeed in the model. In both of these cases, play is always found earlier in youth, and this is in line with our intuition about learning skills in general – the benefits of ability improvements can only be realised if they occur before the activity the skills apply to. There would be no benefit if these skills were learned after foraging and this is why play is found at lower and intermediate reserves, before serious foraging for growth begins. On the other hand, if the benefits of improved ability are delayed until a particular point in time (e.g. sexual maturity, as in Model 3a and 3b), there are no logical benefits from playing earlier or later in youth, and in fact, in both cases, energy security seems to be prioritised over final reproductive performance in the earliest stages of life. In Model 3a, the ability construct directly feeds into the final value and so the results are somewhat less compelling; of course animals play if play is the only way to train for successful reproduction. Here, play is found throughout the model, peaking at both the beginning and end. However, surprisingly, in Model 3b, we still get substantial investment play activity, even when foraging is better practice for reproduction than play (*s*_*f*_ > *s*_*p*_). Here, play starts increasing in the middle of the model, after future energy requirements have been met, and continue until ability reaches its maximum. However, this still makes reasonable sense if the reduced ability gains from playing rather than foraging can be compensated by the reduced predation risk.

The actual ontogenetic course of play is not well mapped across mammalian species. In several species, an inverted‐U‐shaped ontogenetic distribution of play peaking around weaning has been observed (Byers & Walker, 1995). This would correspond to our Model 3b but not to the other Models. It needs to be considered, though, that our models do not start at birth but rather at the age when the young animals become able to forage independently. Thus, the models may not cover very early play.

The models discussed here could be interpreted as applying to three different types of play behaviour, each more appropriate for the learning of adult behaviours in different species. One common categorisation within play activities is between locomotor‐rotational play, object play, and social play (Bekoff & Byers, 1981). The first model presented is perhaps most representative of some kind of prey species, such as a grazing mammal from *Ruminantia* (e.g. a Thomson's gazelle). Here, the focus of their abilities are in predator avoidance, a primary concern for this kind of life history (whereas abilities like foraging success and mating success are possibly less variable and less amenable to improvements in ability). The young of this species often engages in locomotor‐rotational play, which is most similar to the kinds of movements needed for predator avoidance and escape behaviour, including the exuberant stotting behaviour hypothesised to signal their fitness to potential predators. The second model is most representative of a predatory species from a carnivorous taxon such as *Felidae* (e.g. a Wildcat). Cats are known to engage extensively in object‐play and in social quasi‐predatory play (Caro, 1980, 1995), which may offer some practice for serious hunting. Since their foraging success depends on catching prey, there is a much higher variability in success, and one would expect there is plenty of room for improvement in prey‐catching abilities, and perhaps less concern for predator escape responses. Model 3a is most representative of some kind of highly social mammal, such as *Rodentia* (e.g. a Norway rat). Rats forage alone, and so it is almost inconceivable that any abilities relating to the social acts instrumental in reproduction could be learned through this behaviour. Rats also tend to engage in extensive social play (such as rough‐and‐tumble play), which often continues into adulthood (Thor & Holloway, 1984), and could conceivably offer practice for a variety of social activities required to achieve reproduction in their species, including the fighting of other males and general courtship behaviour. Model 3b is perhaps most representative of a social hunter, from taxa such as *Canidae* or *Cetacea* (e.g. a Grey wolf or a Bottlenose dolphin). These species live in groups and are known to forage cooperatively together, and this may plausibly offer some transferable skills that could be applied to the activities of courtship and reproduction.

Two existing models in the literature bear some similarities with the approach we have taken. Auerbach et al. (2015) utilised an agent‐based model involving a population of individuals who could either forage or rest, with a mutant strategy that would sometimes play instead of resting. Counterintuitively they found that if play was frivolous (with no adaptive benefits), play emerged in a small proportion of the population, but this seems to be a consequence of the finite depleting resources being used up more quickly, and the non‐players suffering as a result. Our model differs by instead modelling the optimal behaviour of an individual trying to survive until the end of the time period and we explicitly modelled the ability concept, as a variable that increased probabilistically with each bout of play. Similar to our approach, Grunloh and Mangel (2015) used stochastic dynamic programming to determine when an individual should play (to acquire skills) or to exit the model. In their model, skill‐level (ability) depleted with time, with time being the cost of continuing to play, a feature that could be investigated with extensions to our model. The final fitness function was a function of the final skill‐level (as in our Model 3a and 3b), but did not allow for skills to be instrumental in reducing costs of other behaviours (as in our models 1 and 2). All of these existing modelling approaches provide some insights into the feasibility of the evolution of play, even in cases where the activity has only small, delayed benefits, or no direct benefits at all.

If play does offer practice benefits for adult behaviours as has been widely hypothesised, it does not preclude the possibility that play has multiple functions and may offer other (perhaps more significant) benefits for playing individuals. These other possibilities have been widely discussed and include outcomes such as improved social bonding and cohesion, innovation, self‐assessment, and even the expenditure of excess energy (Baldwin & Baldwin, 1977; Barber, 1991; Fagen, 1981) and multiple benefits of play have been shown to occur within a species (Nunes & Monroy Montemayor, 2023). Additionally, in some cases, play may even have immediate rather than delayed benefits (Palagi, 2023). Moreover, one prominent alternative hypothesis, the Surplus Resource model (Burghardt, 1984, 2005), states that play occurs because of excess resources, such as freely available time and energy, that cannot be put towards any useful serious behaviour, but rather gets expressed as seemingly frivolous activity. These ideas are not mutually exclusive with the Play as Practice hypotheses, rather, the arguments for each are complementary, and these causal levels could be operating in parallel. Our model, which explicitly tracks the energy state of an individual, may offer a useful approach to assess surplus resource ideas alongside other plausible functions, within a single framework.

One important thing to reiterate is what the choice at each timestep represents or can be interpreted as. Depending on the speed of growth and development of the species, a single timestep could be multiple days, or several timesteps may represent a single day. It is unrealistic to suggest that an individual plays or forages continuously over multiple timesteps, rather we can consider the choice at each timestep to represent a small part of the period where the individual has additional unused time to utilise. In this sense, we are modelling what an individual does with its spare time. We consider the remainder of the unmodeled time to include subsistence foraging (enough to exactly balance basal metabolism), daily sleep requirements, and other necessary activities such as grooming. The available time then becomes a choice between playing, additional rest, and additional foraging for reserve building and growth purposes.

In these models, we have primarily considered foraging as the option for ‘serious’ behaviour, and for many species, energy balance is the key life history variable that activity is focussed towards. However, the energetic reserve variable, *x*, could just as easily be reinterpreted as some kind of individually‐owned resource such as reputation, dominance relation or territorial control, which could be increased with an appropriate corresponding activity, depletes with time and can be turned into a fitness payoff at the end of the model. The ability *a* could represent any skill that cannot itself be directly turned into fitness but lasts without depreciation and enhances the rate or probability of resource acquisition or reduces the risk of dying. With some careful consideration of the parameter values and the rules for updating the variables, the basic structure of the model can be maintained whilst reinterpreting the behaviours within. Therefore, this class of models may be substantially more general than the simple choice between foraging, play and rest.

One other feature of our model that should not go undiscussed is the terminal value function. With the default parameters, models 1 and 2, this is a binary outcome, with a success (value = 1) when energy reserves are above *x*_crit_ and a failure (value = 0) when below, and models 3a and b have the additional feature that makes the final payoff a linear function of ability. These are very simple value functions and probably unrealistic in nature, since excess reserves over and above *x*_crit_ would, in many species, lead to even greater future reproductive success (as more energy could be put into producing milk, used for securing higher quality mates or gaining a better resourced territory for reproduction). For these reasons, we specified one parameter (*q*) as an exponent of final energy reserves. Through our sensitivity analysis, we found that for models 1, 2 and 3a, similar amounts of play is expected, regardless of this final value transformation. The exception to this is Model 3b, where play becomes very rare if more energy has more value (since in this version of the model, foraging feeds directly into final value through both greater energy and ability).

Outside of the exploration of the parameter space within this model specification, there are a vast number of different variations of this basic model, with slightly different mechanics (alternative variable relationships and rules for updating). We have explored a number of these at a shallower level of analysis, with similar results. Unfortunately (and perhaps for the benefit of the reader), both time and space does not permit us to explore the details of each of these. Since play is a rich and diverse phenomenon, with many plausible delayed benefits, each of these models are just one of many possible ways to set‐up the decision problem. We have only selected a few as representative cases among many that were explored. In this set of models, we have only considered one construction of the ability concept, where ability linearly changes the value of some probability parameter of the model (*m*_*f*,_ *y*_*z*,_ or *ω*_*z*_), and the ability variable itself changes by 1 unit for every improvement. In reality, different activities or attributes of a task may have radically different learning curves, and we might expect this to affect the marginal improvements that further practice would offer.

Further limitations to our model include difficulties in fitting the model to a specific species using precise parameters. This is in part due to dynamic programming approach we have used, which means a great number of abstractions and simplifications needed to be made. This generality can be both a strength and a weakness, showing the phenomena we might expect to see in all animals that live under certain environmental constraints, and at the same time, limiting the ability of that model to make specific predictions about a certain species.

### 3.1 The practicality of play as practice

This set of models explores choices between three activities, one of which, with no other benefits, increases ability, an instrumental variable which can improve performance of other activities. We have labelled this activity as play, despite there being nothing in the model that means this needs to be interpreted as play, since it could equally be thought of as specific training, directed practice or more general preparatory exploration of the local environment (‘ability’ conceived as better knowledge of the landscape). Although this model shows plausible situations where play would be beneficial when the alternatives are only foraging or resting, it cannot demonstrate how or why play is better practice than other more efficient forms of training, since “it is not necessary to play in order to practise – there is no reason why the animal should not just practise” (Loizos, 1967, p.185).

Firstly, we might assume that playing is often less efficient practice for a given activity than the activity it mimics. One real fight is likely many times worth the value of a play fight, in terms of actual learning potential. Pretend or simulated aquatic locomotion may be no match for being dumped into a body of water and being allowed to sink or swim. When mistakes are costly, time is pressured and the problem or task being tackled is real, rather than abstract, we might expect our abilities to improve much more quickly. After all, “the world is its own best model” (Brooks, 1991). Our model shows one solution to this problem: even if doing the serious activity itself is better training than practising through play, if the costs of mistakes in the real world are high enough, it may be better to prepare one's abilities beforehand, rather than die or get seriously injured on the initial stages of a steep learning curve.

However, this does not solve the root of the problem. Why do animals play, rather than deliberate and direct practice towards the very specific movements needed for whatever activity it is mimicking? Play is often much more diverse, exploratory, exuberant, and exaggerated than its serious counterpart behaviours. Deliberate practice on the other hand is often based on repetition, deviating as little as possible from the ultimate movements they imitate. The ‘wax‐on, wax‐off’ and fence painting activities in the fictitious story, *The Karate Kid*, unbeknownst to the protagonist, were precisely the movements he needed to win the fight at the story's conclusion. Likewise, the modern drilling methods of world class sports teams and athletes offer technique‐ and tactic‐specific improvements in ability within far shorter periods of time than any playful imitation could offer.

We can only speculate on the reasons. Firstly, we may be mistaken in how much play actually deviates from the movements and behavioural sequences they train for. An ideal, most efficient prey‐catching, escaping or dominance‐winning serious movement may be much more streamlined than its playful counterpart. However, in real life such movements can hardly be accomplished along the ideal trajectories because the world rarely provides ideal conditions or submits passively to the action of the animal. Substrates are often tricky for movements and recipients of the intended behaviours often actively resist and counteract. Perhaps the exaggerated and exuberant nature of playing attempts to train for the natural variation in the serious situation and real problems that require practice need more flexible and less stereotypic movements for success (Špinka et al., 2001). It may only be for highly repetitive, non‐varying tasks that specific deliberate practice may be preferable.

Secondly, because of its highly variable nature, play may offer training across more than one ability at the same time. Whilst repeated motor practice on a specific task may be the best way to improve muscle abilities for that specific movement, play, by introducing variability, may help general abilities on a wider range of motoric, cognitive, and affective abilities, including reaction‐time, visual assessment, decision‐making speed, and the handling of unexpected events (Špinka et al., 2001).

Thirdly, it is possible that play helps the individual explore the space of potential actions far more widely than directed practice. In this way, play may be a rich source of behavioural innovation (Bateson & Martin, 2013). For individuals encountering completely novel situations or needing to perform novel actions, then experience with similar scenarios may be advantageous. Deliberate practice is probably the best approach for commonly and repeatedly found scenarios, but in everyday activities, there are frequently many different rarely encountered scenarios. Play might work by generating opportunities to solve rare problems that would never be encountered through deliberate practice. It is no wonder that many researchers consider play to be a form of exploration: an exploration of possible means, with little concern for the ends (Bruner, 1972; Miller, 1973), and with a changing emphasis “…from the question of ‘what does this object do?’ to ‘what can *I* do with this object?’” (Hutt, 1966, p.76). Future models should attempt to address this key question regarding the benefits of play over deliberate practice and training.

## AUTHOR CONTRIBUTIONS

**Dave E. W. Mallpress:** Conceptualization (equal); formal analysis (equal); methodology (lead); software (lead); visualization (lead); writing – original draft (lead); writing – review and editing (equal). **Marek Špinka:** Conceptualization (equal); formal analysis (equal); funding acquisition (lead); writing – review and editing (equal).

## CONFLICT OF INTEREST STATEMENT

We declare no conflicts of interest.

## Data Availability

No data was collected in this study. The analytic models were coded in MATLAB and all source code is available on the Open Science Framework and can be accessed at: https://osf.io/yrupq/?view_only=e86ca97ad7634a438bab5ddd6ab2ecdd.

## APPENDIX A

### A.1 Technical details of Model 1 (play as increasing predator avoidance)

The final evaluation function depends on the level of energetic reserves available at the end of the time period, *T*. Final ability has no effect on this terminal reward, so for all values of *a*:

(1)
$$U^{*}\left(x,a,T\right)=\left\{\begin{array}{cc} x^{q} & \;\text{if}\;x\geq x_{\text{crit}}\\ 0 & \;\text{if}\;x<x_{\text{crit}} \end{array}\right.$$

The parameter *q* is an exponent that changes the shape of the value function. (Note: For 0 < *q* < 1, the value function would be decelerating, *q* = 1, linear, and *q* > 1 accelerating). We have set the default of this to *q* = 0, making *U* ^***^(*x*,*a*,*t*) = 1 for all cases where *x* ≥ *x*_crit_. We have kept this as a simple all‐or‐nothing final value, where excess reserves at time *T* do not add value, but added the parameter anyway, so that in the sensitivity analysis we can explore the effect of alternative value functions.

If the reserves fall to zero at any point during the animal's life, the animal dies of starvation and its reproductive value is zero. For all values of *a* and *t*:

(2)
$$U\left(0,a,t\right)=0$$

For this model, it would be convenient to store any probabilistic state changes in matrices; the first column representing the state change, and the second representing the probability of the change. The possible gains in energy associated with parental provisioning are given by ***β***.

When *t* ≤ *τ*, for any activity: and when *t* > *τ:*

(3)
$$\beta =\left[\begin{array}{cc} w_{e} & w_{z}\\ 0 & 1-w_{z} \end{array}\right]\;\;\;\;\;\;\;\;\;\;\;\;\;\;\;\;\;\;\;\;\;\;\beta =\left[\begin{array}{cc} 0 & 1\\ 0 & 0 \end{array}\right]$$

The possible gains in energy associated with the activity of foraging are given by ***γ***.

(4)
$$\gamma =\left[\begin{array}{cc} y_{e} & y_{z}\\ 0 & 1-y_{z} \end{array}\right]$$

The possible improvements in ability depend on the activity and are given by the ***α*** matrices.

(5)

The mortality rate from predation when foraging (*m*_*f*_) changes as a function of ability. When ability is 0, the mortality rate when foraging is at its highest. This decreases linearly with improved ability – when ability is at its maximum (*A*), the predation rate whilst foraging is equivalent to the predation rate whilst playing.

(6)
$$m_{f}\left(a\right)=m_{p}+\left(1-\frac{a}{A}\right)\left(m_{f}\left(0\right)-m_{p}\right)$$

#### A.1.1. The dynamic programming equations

The next step is to calculate the values of each action in different states. Starting with *t* = *T*‐1, for each state *x* = [1, *X*], *a* = [0, *A*], we can calculate the value of foraging by summing up the value of all possible final states *x*,*a* at *T* that the individual could end up, multiplied by the probability of landing in each of those states. This gives us a value of foraging *U*_*f*_ at *T*‐1:

(7)
$$\begin{array}{cc} U_{f}\left(x,a,t\right)= & \sum_{i=1}^{2}\sum_{j=1}^{2}\sum_{k=1}^{2}\beta_{i,2}\gamma_{j,2}{\alpha^{f}}_{k,2}\times \\ & U^{*}\left(x-c_{f}+\beta_{i,1}+\gamma_{j,1},a+{\alpha^{f}}_{k,1},t+1\right)\times \left(1-m_{f}\left(a\right)\right) \end{array}$$

Since the individual can never have more energy than *X*, or less than 0, nor more ability than *A*, any state variable that exceeds those limits are treated as being at that limit.

The same value calculation can be done for play:

(8)
$$U_{p}\left(x,a,t\right)=\sum_{i=1}^{2}\sum_{k=1}^{2}\beta_{i,2}{\alpha^{p}}_{k,2}\times U^{*}\left(x-c_{p}+\beta_{i,1},a+{\alpha^{p}}_{k,1},t+1\right)\times \left(1-m_{p}\right)$$

And for resting:

(9)
$$U_{r}\left(x,a,t\right)=\sum_{i=1}^{2}\beta_{i,2}\times U^{*}\left(x-c_{r}+\beta_{i,1},a,t+1\right)\times \left(1-m_{r}\right)$$

Now we have a value for each activity at *t* = *T*‐1. We can then calculate the optimal value *U**(*x,a,t*), by taking the maximum for each of these activity‐specific value functions.

(10)
$$U^{*}\left(x,a,t\right)=\max\left(U_{f}\left(x,a,t\right),U_{p}\left(x,a,t\right),U_{r}\left(x,a,t\right)\right)$$

We can also specify a choice function, Ψ, which is the optimal activity for any given state:

(11)
$$\Psi \left(x,a,t\right)=\left\{\begin{array}{ccc} \text{forage} & \text{if} & U^{*}\left(x,a,t\right)=U_{f}\left(x,a,t\right)\\ \text{play} & \text{if} & U^{*}\left(x,a,t\right)=U_{p}\left(x,a,t\right)\\ \text{rest} & \text{if} & U^{*}\left(x,a,t\right)=U_{r}\left(x,a,t\right) \end{array}\right.$$

We can iterate through this procedure (7–11) for *t*‐2, and then *t*‐3, and so on until *t* = 0. This is the dynamic programming equations that determine the value of the choices the individual can make, assuming that all future choices are optimal.

#### A.1.2. The forward model

We first specify a probability function, *Θ*, that calculates the proportion of individuals within a population ending up in each next state, given their current state. This is mathematically equivalent to the probability of any single individual ending up in any given state.

Starting with *Θ*(*x*_start_, *a*_start_, 0) = 1 (and all other states being equal to 0), we find the corresponding optimal action *Ψ*(*x*_start_, *a*_start_, 0). From here, given action *Ψ*, we find the probability of each possible next state at *t* = 1.

If *Ψ*(*x*,*a*,*t*) **=** *forage*, then for each value of *i*, *j* and *k*:

(12)
$$\begin{array}{cc} \Theta \left(x-c_{f}+\beta_{i,1}+\gamma_{j,1},a+{\alpha^{f}}_{k,1},t+1\right)= & \Theta \left(x-c_{f}+\beta_{i,1}+\gamma_{j,1},a+{\alpha^{f}}_{k,1},t+1\right)+\\ & \left(\Theta \left(x,a,t\right)\beta_{i,2}\gamma_{j,2}{\alpha^{f}}_{k,2}\left(1-m_{f}\left(a\right)\right)\right) \end{array}$$

If *Ψ*(*x*,*a*,*t*) **=** *play*, then for each value of *i*, and *k*:

(13)
$$\begin{array}{cc} \Theta \left(x-c_{p}+\beta_{i,1},a+{\alpha^{p}}_{k,1},t+1\right)= & \Theta \left(x-c_{p}+\beta_{i,1},a+{\alpha^{p}}_{k,1},t+1\right)+\\ & \left(\Theta \left(x,a,t\right)\beta_{i,2}{\alpha^{p}}_{k,2}\left(1-m_{p}\right)\right) \end{array}$$

and if *Ψ*(*x*,*a*,*t*) **=** *rest*, then for each value of *i*:

(14)
$$\Theta \left(x-c_{r}+\beta_{i,1},a,t+1\right)=\Theta \left(x-c_{r}+\beta_{i,1},a,t+1\right)+\left(\Theta \left(x,a,t\right)\beta_{i,2}\left(1-m_{r}\right)\right)$$

We can also calculate the proportion of individuals who are dead at time *t*, by subtracting the sum of the proportion of all living individuals at *t*:

(15)
$$\Theta \left(\text{dead},t\right)=1-\sum_{x=1}^{X}\sum_{a=0}^{A}\Theta \left(x,a,t\right)\;$$

At *t* = 1, we iterate through all values of *x* and *a*, applying the relevant calculations depending on the choice *Ψ*(*x*,*a*,*t*). This is repeated for *t* = 2,3…etc. until *t* = *T*.

### A.2 Technical details of Model 2 (play as increasing foraging success)

#### A.2.1. Modifications to specification

Foraging success probability (*y*_*z*_) is modified to make it a linearly increasing function of ability.

(16)
$$y_{z}\left(a\right)=y_{z}\left(0\right)+\left(\frac{a}{A}\right)\left(y_{z}\left(A\right)-y_{z}\left(0\right)\right)$$

This new relationship needs to be fed into the matrix storing state changes, modifying Equation (4) as follows.

(17)
$$\gamma \left(a\right)=\left[\begin{array}{cc} y_{e} & y_{z}\left(a\right)\\ 0 & 1-y_{z}\left(a\right) \end{array}\right]$$

And also a minor change in the equation for the value of foraging (7)

(18)
$$\begin{array}{cc} U_{f}\left(x,a,t\right)= & \sum_{i=1}^{2}\sum_{j=1}^{2}\sum_{k=1}^{2}\beta_{i,2}\gamma {\left(a\right)}_{j,2}{\alpha^{f}}_{k,2}\times \\ & U^{*}\left(x-c_{f}+\beta_{i,1}+\gamma {\left(a\right)}_{j,1},a+{\alpha^{f}}_{k,1},t+1\right)\times \left(1-m_{f}\right) \end{array}$$

The default parameters for the second model are all listed in Table 1 (main text). The remaining dynamic programming equations are the same (8–11). For the forward model, Equation 12 is almost the same, replacing *m*_*f*_(*a*) with *m*_*f*_ and *γ* with *γ*(*a*), and all other calculations are identical (13–15).

### A.3 Technical details of Model 3 (play as increasing reproductive success)

#### A.3.1. Modifications to specification

For the third model, we now make the final time *T* explicitly the point of sexual maturity, and the final score, *U**(*x,a*,*T*) directly related to reproduction. Here, we make the reproduction payoff a function of both final energy reserves (*x* at time *T*) and final ability (*a* at time *T*).

We designate *ω*_*z*_ the probability of success when trying to reproduce and make it a linearly increasing function of ability, with *ω*_*z*_(0) as the baseline success probability with no ability, and *ω*_*z*_(A) as the maximum success probability when ability is at a maximum:

(19)
$$\omega_{z}\left(a\right)=\omega_{z}\left(0\right)+\left(\frac{a}{A}\right)\left(\omega_{z}\left(A\right)-\omega_{z}\left(0\right)\right)$$

As before, play increases ability with some probability (*s*_*p*_ > 0). For Model 3a, we remove the ability benefits of foraging (*s*_*f*_ = 0) and for Model 3b, we retain them (*s*_*f*_ > *s*_*p*_).

We then set the final value function so that only if the energy reserves are above the critical threshold for reproduction, the individual gets a payoff. The payoff is a product of the probability of success function of ability, *ω*_*z*_(*a*), and an exponent of the reserves, *x* ^*q*^, where 0 ≤ *q*:

(20)
$$U^{*}\left(x,a,T\right)=\left\{\begin{array}{cc} x^{q}\times \omega_{z}\left(a\right) & \;\text{if}\;x\geq x_{\text{crit}}\\ 0 & \;\text{if}\;x<x_{\text{crit}} \end{array}\right.$$

All other parameters are the same as in Table 1. The dynamic programming equations for the activities (Equation (7), (8), (9)) are the same, except that the mortality associated with foraging is no longer a function of ability.

### A.4 Sensitivity analyses

For each of the models, we performed a one‐at‐a‐time sensitivity analysis for each of the key parameters of the model. For this, we varied a single parameter across a wide range of values (including extreme and unrealistic values to show the cases where the model breaks down). We ran the dynamic programming algorithm for each parameter setting to discover the optimal choice function. From that, we then ran the model forward to find the proportion of optimal individuals ending up in each part of the state space and what their activity choice would be. We then totalised the proportion of each activity over the lifetime, and then finally plotted the proportion of each activity against each value in the parameter range.

The parameter ranges we used are listed below:

**Table:**

*m*_*f*_	0.0001 ≤ *m*_*f*_ ≤ 0.3
*m*_*p*_	0.0001 ≤ *m*_*p*_ ≤ 0.06
*m*_*r*_	0.00001 ≤ *m*_*r*_ ≤ 0.06
c_f_	0 ≤ *c*_*f*_ ≤ 5
*c*_*p*_	0 ≤ *c*_*p*_ ≤ 14
*c*_*r*_	−1 ≤ *c*_*r*_ ≤ 3
*s*_*f*_	0 ≤ *s*_*f*_ ≤ 1
*s*_*p*_	0 ≤ *s*_*p*_ ≤ 1
*w*_*e*_	0 ≤ *w*_*e*_ ≤ 10
*w*_*z*_	0 ≤ *w*_*z*_ ≤ 1
*y*_*e*_	1 ≤ *y*_*e*_ ≤ 10
*y*_*z*_	0 ≤ *y*_*z*_ ≤ 1
*x*_start_	1 ≤ *x*_start_ ≤ 280
*a*_start_	0 ≤ *a*_start_ ≤ 95
*τ*	1 ≤ *τ* ≤ 100
*x*_crit_	1 ≤ *x*_crit_ ≤ 265
*q*	0 ≤ *q* ≤ 2
*ω*_*z*_(0)	0 ≤ *w*_*z*_(0) ≤ 1
*X*	150 ≤ *X* ≤ 600
*A*	10 ≤ *A* ≤ 250
*T*	50 ≤ *T* ≤ 350

### A.5 Model 1 (play as increasing antipredator ability)

From the sensitivity analysis, we can see that as the initial probability of predation mortality, *m*_*f*_(0), decreases, so does the propensity to play, since little benefits are gained by playing if foraging is not much more dangerous than playing. Similarly, if the predation risk of playing, *m*_*p*_, increases too much, it is no longer performed in the ‘safe space’ which gives play the only clear benefit it has in this model. The reason why the proportion dead increases, even when the individual is not playing at all is that the minimum mortality rate from foraging is pegged to the mortality rate of play, *m*_*f*_(*A*) = *m*_*p*_ and so the mortality risk of play sets the lower limit on the mortality risk of the most able forager. Unsurprisingly, the mortality risk of rest, *m*_*r*_, only really effects the time spent resting. Counter‐intuitively, as the cost of foraging, c_f_, increases, the time spent playing decreases. This makes sense if we consider that play is energetically expensive, and so to recoup any excess energy spent on playing, considerably more low‐profitability foraging must be performed (and thus the benefits of play are lost). As might be expected as the energy cost of play, *c*_*p*_, decreases or the energy cost of rest, *c*_*r*_, increases, the time spent playing increases. More interestingly, play activity is still maintained at quite high levels even when play has a high energy cost (*c*_*p*_ = 5).

The figures for the parentally provisioned amount, *w*_*e*_, and probability, *w*_*z*_, the starting reserves, *x*_start_, and the time where parental provisioning stops, *τ*, have almost identical shapes in terms of proportion of time spent for each activity (at least over the range some of the parameter range). This is because they are functionally equivalent, as increasing any of them decreases the probability of starving or failing to reach the critical threshold and increases the likely amount of reserves left after the juvenile period. The propensity to play is lower when this energy buffer provided by the parent is absent (if *w*_*e*_, *w*_*z*_, or *x*_start_ is close to zero), or when there is little initial energy (*x*_start_ is low). This is because the individual should be more concerned with foraging immediately (and ‘learning on the job’, so to speak) than increasing ability through the indirect but safer (play) route. Similarly, when these parameters are high, play is absent, since little future foraging is necessary to reach the terminal time above the critical energy threshold and the individual can spend most of their time resting. The figure for the critical reserves level *x*_crit_ is a mirror image of that for *x*_start_ since it just involves the movement of the start and finish points and the energetic trajectory that needs to be taken.

### A.6 Model 2 (play as increasing foraging success ability)

Many of the patterns of predicted activity between Models 1 and 2 are very similar and can be accounted for by similar reasoning (e.g *m*_*f*_, or the equivalence of *w*_*e*_, *x*_start_, and *τ*). Play is only found when the mortality rate of play, *m*_*p*_, is very low, and is reaches its highest proportion of time spent when the energetic cost is lower. If this second model, as suggested in the main text, is most representative of some kind of object play, we may expect much lower energetic costs than with locomotor‐rotational or boisterous social play. The presence of play in this model is considerably more fragile for many parameter settings and often falls to zero very rapidly for parameter variations outside the default settings (see Table 1, main text), making it the least robust of all the models presented here. Model 2 is also the most dependent on the availability of parental provisioning, without which play is entirely extinguished. Additionally, play is only found when the initial ability for foraging success, *y*_*z*_(0) and *a*_start_, is particularly low, and when rest is more dangerous (*m*_*r*_).

Quite intuitively, play increases, when the probability of increasing ability from play, *s*_*p*_, increases – when the benefits of play are greater, we would expect to see more play. Similarly, when the probability of increasing ability from foraging, *s*_*f*_, is very low, then play is the most efficient way to improve ability and so the proportion of time spent on play is highest. Unexpectedly, the proportion of play drops to zero for 0.3 < *s*_*f*_ < 0.5, but then increases again when foraging is effective practice.

### A.7 Model 3a (play as increasing reproductive ability): *s*_*f*_ *=* 0

In Model 3a, since play is a direct contributor to the final value function, play is found in almost all parameter settings. Play disappears when the mortality rate from foraging, *m*_*f*_, is very high (since without foraging, the individual will die of starvation, and so most individuals die very early by chance). Likewise, play also disappears when the mortality rate from play, *m*_*p*_, is high, in which case, play is simply too risky to engage in (and most individuals end up dead). However, play is found at reasonably high levels, even when the energetic costs of play, *c*_*p*_, are very high. The proportion of time spent in play is almost entirely independent of the probability of ability improvements from play, *s*_*p*_, (with the exception of when *s*_*p*_ = 0), since all ability improvements directly contribute to final reproductive value and any excess energy is worthwhile utilising. More interestingly, and inspiring our inquiry into Model 3b, play is still found even for some cases where *s*_*f*_ > *s*_*p*_, where foraging is doubly beneficial towards the final value. In this case, the reduced mortality risks associated with play must compensate for the inferior ability improvements.

### A.8 Model 3b (play as increasing reproductive ability): *s*_*f*_ = 0.8

In Model 3b, play vanishes fairly quickly as the mortality rate associated with the activity of play (*m*_*p*_) increases. Here, the small increase in the probability of reproduction cannot compensate for the probability of dying from playing in the first place. As in Model 3a, as the energetic cost of play (*c*_*p*_) increases to high levels, play is still maintained at a significant level. Counterintuitively, as the cost of rest (*c*_*r*_) increases, rest increase and play decreases. As provisioning and foraging amounts and probabilities (*w*_*e*_, *w*_*z*_, *y*_*e*_, *y*_*z*_), as well as provisioning duration (*τ*) and initial reserves (*x*_start_), increase the proportion of time spent playing increases. This is intuitive, as when energy becomes more abundant, an individual can spend this excess energy on gaining ability in a safer environment.
